# Hypogonadism and Sexual Dysfunction in Testicular Tumor Survivors: A Systematic Review

**DOI:** 10.3389/fendo.2019.00264

**Published:** 2019-05-07

**Authors:** Sandro La Vignera, Rossella Cannarella, Ylenia Duca, Federica Barbagallo, Giovanni Burgio, Michele Compagnone, Andrea Di Cataldo, Aldo E. Calogero, Rosita A. Condorelli

**Affiliations:** ^1^Section of Endocrinology, Department of Clinical and Experimental Medicine, University of Catania, Catania, Italy; ^2^Unit of Pediatric Hematology and Oncology, Department of Clinical and Experimental Medicine, University of Catania, Catania, Italy

**Keywords:** hypogonadism, testicular tumor, testosterone, sexual dysfunction, cardiovascular risk

## Abstract

Testicular tumor is the most common malignancy in men of reproductive age. According to the tumor histology and staging, current treatment options include orchiectomy alone or associated with adjuvant chemo- and/or radiotherapy. Although these treatments have considerably raised the percentage of survivors compared to the past, they have been identified as risk factors for testosterone deficiency and sexual dysfunction in this subgroup of men. Male hypogonadism, in turn, predisposes to the development of metabolic and cardiovascular impairment that negatively affects general health. Accordingly, longitudinal studies report a long-term risk for cardiovascular diseases after radiotherapy and/or cisplatin-based chemotherapy in testicular tumor survivors. The aim of this review was to summarize the current evidence on hypogonadism and sexual dysfunction in long-term cancer survivors, including the epidemiology of cardiovascular and metabolic disorders, to increase the awareness that serum testosterone levels, sexual function, and general health should be evaluated during the endocrinological management of these patients.

## Introduction

The testicular tumor is the most common solid malignancy in young adult men (aged 14–44 years) in Western countries and represents ~1.5% of all tumor diagnosis worldwide (1). Its incidence has risen over the last decades, especially in industrialized countries (2). Testicular tumor affects from <1 per 100,000 males in many African and Asian nations to >9 per 100,000 men in the highest-incidence areas of Northern and Western Europe. Despite the highest incidence in more developed countries and particularly in Europe, the incidence-to-mortality ratio is 26:1 in northern Europe compared with 2:1 in Southeast Asia, South–Central Asia, and Africa. This indicates the need to improve the treatment strategy in some non-European countries (3).

Over the years, a multitude of classifications have been proposed for testicular tumor, reflecting a progressive better understanding of its pathogenesis. Despite the testis being a relatively small organ, it consists of many different cell types; thus, it may give rise to a large variety of neoplasms (Table 1). Nonetheless, more than 95% of testicular tumors are testicular germ-cell tumors (TGCTs) derived from germ cells (4). Sex cord stromal tumors and other non-germ-cell tumors are exceedingly rare. The most recent WHO classification, which has been published in 2016, represents a transition from an exclusively morphological system into one that takes into account the histological composition, the age of onset, and the pathogenic mechanisms of testicular tumor development (5). This new classification recognizes two major types of TGCTs: those derived from germ-cell neoplasia *in situ* (GCNIS) and those unrelated to GCNIS (5).

**Table 1:** Classification of testicular tumors.

**Testicular Tumors**	**Germ-Cell Tumor**	**GCNIS-derived**	**Type II**	**Seminoma**	
				**Non-seminoma**	• Yolk sac tumor • Embryonal carcinoma • Teratoma, post-pubertal type • Choriocarcinoma
		Non GCNIS-derived	Type I	• *Yolk sac tumor, pre-pubertal type* • *Teratoma, pre-pubertal type*	
			Type III	• *Spermatocytic tumor*	
	Sex Cord/Stromal Tumor	Leydig cell tumor	• *Malignant Leydig cell tumor*		
		Sertoli cell tumor	• *Malignant Sertoli cell tumor* • *Large cell calcifying Sertoli cell tumor* • *Intratubular large cell hyalinizing Sertoli cell neoplasia*		
		Granulosa cell tumor	• *Adult Type* • *Juvenile Type*		
		Techoma/fibroma			
		Others	• *Myoid gonadal stromal tumor* • *Mixed* • *Unclassified*		
	Germ-cell and sex cord/gonadal stromal tumors	Gonadoblastoma			
		Unclassified			
	Miscellaneous	Hemangioma			
		Hematologic neoplasms			
		Secondary tumors			
		Ovarian epithelial tumors			
		Tumors of the collecting ducts and rete testis			
	Paratesticular tumors	Adenomatoid tumor			
		Mesothelioma			
		Epididymal tumor			
		Soft tissue tumors	*Lipoma and Liposarcoma Leiomyoma and Leiomyosarcoma Fibroblastic and Myofibroblastic Tumors*		

*GCNIS, germ-cell neoplasia in situ*.

Management of testicular tumor is controversial. After orchiectomy, subsequent management options include active surveillance, adjuvant chemotherapy or radiotherapy, and primary retroperitoneal lymphadenectomy (RPLND) (6). Treatment-related toxicity is crucial considering that the long-term survival rate of TGCTs is ~99%, regardless of treatment strategy (6). For this reason, the most recent guidelines focus on minimizing unnecessary treatments to avoid adverse effects that are associated with them and to customize treatment for each patient considering patient's individual risks and his individual wishes (7). Each patient should be informed about the potential advantages and disadvantages of surveillance and adjuvant therapy (7). While surveillance allows most patients to avoid additional treatment, adjuvant therapy significantly lowers the relapse rate (7). Over the years, enthusiasm for adjuvant radiotherapy has been markedly reduced by the risk of radiation-induced secondary cancers. An increasing evidence suggests that active surveillance post-orchiectomy is a suitable alternative to adjuvant regimens in both stage I seminomas and non-seminomas (6). In the treatment of advanced testicular tumor, the current standard of care includes the use of platinum-based chemotherapy [bleomycin, etoposide, and cisplatin (BEP)] (6). A clear dose relationship has been established for the following BEP sequelae: pulmonary toxicity, fertility (8), neurotoxicity, ototoxicity, nephrotoxicity, metabolic syndrome, and hypogonadism (9, 10).

Hypogonadism has been often reported in testicular tumor survivors. Indeed, testicular tumor may represent a feature of the so-called testicular dysgenesis syndrome (TDS) (11, 12). The possibility exists that TDS may somehow impair Leydig cell function. Accordingly, studies indicate that germ-cell malignancy itself may be associated with poorer gonadal function in the remaining testis prior to other treatments (13). Also, the occurrence of microlithiasis (a feature of TDS) in the remaining testis has been shown to predict the incoming of hypogonadism in testicular tumor survivors (14). In addition, because of the radio- and/or chemo-induced Leydig cell damage, adjuvant therapy rises the risk of hormonal deterioration that results in increasing serum luteinizing hormone (LH) levels and decreasing serum testosterone concentrations (15, 16).

Therefore, the aim of this review was to gather together the current evidence of hypogonadism and sexual dysfunction in long-term testicular tumor survivors, including the epidemiology of cardiovascular and metabolic disorders, to increase the awareness to evaluate serum testosterone, sexual function, and general health in testicular tumor survivors.

## Methods

We performed a comprehensive review of the literature aimed at evaluating the occurrence of hypogonadism and its related complications, including cardiovascular, metabolic and bone mineralization impairment, and sexual dysfunction in testicular tumor survivors. A systematic search was made through PubMed, MEDLINE, Cochrane, Google Scholar, and Scopus databases. Data were independently extracted by RC and FB. The search strategy was based on the following keywords: “testicular cancer,” “testicular tumor,” “testosterone,” “hypogonadism,” “cardiovascular,” “diabetes,” “bone,” “osteoporosis,” “erectile dysfunction,” “premature ejaculation,” and “sexual dysfunction.” Additional manual searches were made using the reference lists of relevant studies.

No language restriction was used for any literature search. Information on the year of publication, country, continent, study design, and mean age of patients was collected. Studies that met the following inclusion criteria were included in the qualitative synthesis:

- Full-length articles (including longitudinal, retrospective, cross-sectional, case–control studies, review, and meta-analysis) published between 1990 and 2019;
- Studies carried out on patients with testicular tumor of any histological type and stage, whose treatment (surgery, radiotherapy, and/or chemotherapy) was clearly reported;
- Studies having at least one among gonadotropins, total testosterone, cardiovascular health, metabolic profile, or bone mineralization as main outcome, collected at baseline and or at the follow-up counseling.

Studies that did not met the above-mentioned inclusion criteria were excluded.

## Hypogonadism

Several longitudinal studies have been carried out to assess the Leydig cell function in testicular tumor survivors. The evidence suggests the vulnerability of Leydig cells to platinum-based chemotherapy and radiotherapy. Animal studies have shown Leydig cell apoptosis (as well as in Sertoli and germ cells) induced by cisplatin both on single administration and on a cumulative manner (17–19). In addition, patients receiving more than 20-Gy dose of radiation at the testicular level need testosterone replacement therapy after 15 years of follow-up, as for a half of patients receiving 16-Gr dose of radiation (20). Interestingly, infra-diaphragmatic radiotherapy when administered at the dose of 30 Gy, corresponding to 0.09–0.32 Gy testicular irradiation (21), is associated also with a slightly greater risk for developing testosterone deficiency, according to a study of meta-analysis (22). These findings suggest that Leydig cells are susceptible to minimal irradiation doses (22, 23).

A number of studies compared chemotherapy-, radiotherapy-, and orchiectomy-alone-dependent toxicity. The results are influenced by the length of follow-up, since those having a longer time of surveillance allow drafting of conclusions on the Leydig cell functional reserve. A summary of the risk of developing hypogonadism in testicular tumor survivors is reported in Table 2.

**Table 2:** Summary of available data from studies on hypogonadism in testicular cancer survivors.

**References**	**Study design**	**Total sample**	**Time of enrollment/Follow-up**	**Results**
Nord et al. (13)	Cross-sectional	1,235 patients and 200 controls	11 years	• No difference in testosterone level was found • Higher age-adjusted LH levels vs. controls • Age-adjusted ratio for hypogonadism = 3.8
Huddart et al. (24)	Case–control	680 patients	>5 years post-treatment	• Hypogonadism was more common in patients treated with chemotherapy plus radiotherapy (37%) vs. those treated with orchiectomy alone (6%) (*p* < 0.01) • High LH levels were found in 11% of patients treated with radiotherapy and in 10% of those treated with chemotherapy (*p* < 0.01 vs. orchiectomy alone) • Compared to baseline, a fall in testosterone levels was observed in patients treated with chemotherapy
Eberhard et al. (14)	Case–control	143 patients and 916 age-matched controls	0, 6, 12, 24, 36, and 60 months after therapy	• Chemotherapy and radiotherapy were both associated with risk for hypogonadism at T0, T6, and T12 • Microlithiasis predicted hypogonadism at all time points • Hypogonadism at T0 predicted the risk for hypogonadism at T6, T12, T24, and T36
Sprauten et al.(10)	Prospective	307 patients	18 years	• A significantly higher risk for low testosterone and high LH was found
Bandak et al. (22)	Meta-analysis	1,187 patients treated with chemotherapy and 671 patients treated with orchiectomy from 11 studies; 301 patients treated with chemotherapy plus non-conventional therapy and 531 patients treated with orchiectomy from 7 studies; 761 patients treated with radiotherapy and 494 patients treated with orchiectomy from 6 studies	1–12 years	• Compared to orchiectomy alone, risk for hypogonadism was significantly higher in chemotherapy (OR 1.8), non-conventional therapy (OR 3.1), and infradiaphragmatic radiotherapy (OR 1.6)
Kerns et al. (25)	Cross-sectional	1,214 patients treated with chemotherapy or post-chemotherapy RPLND	4.2 years post-treatment (range: 1 to 30 years)	• Hypogonadism occurs in 10.2% of patients

*LH, luteinizing hormone; OR, odds ratio; RPLND, retroperitoneal lymph node dissection; T, time*.

A prospective multicenter study on 1,235 testicular tumor survivors (mean age 44 years) investigated the risk for hypogonadism after a 11-year-long follow-up. While no difference in serum testosterone levels was found among patients and controls (*n* = 200), age-adjusted LH levels were higher in the former. In greater detail, the age-adjusted OR of hypogonadism was 3.8 in testicular tumor survivors and showed to increase with treatment intensity being marginally high for surgery alone, 3.5 for radiotherapy, and 4.8 and 7.9 for low- and high-dose chemotherapy, respectively (13). These findings suggest the occurrence of an age-dependent deterioration in Leydig cell function of testicular tumor survivors, with a higher effect of chemotherapy compared to radiotherapy.

A longitudinal cohort study on 307 patients with testicular tumor reported lower testosterone levels at all surveillance time points, which were done after a mean of 9 years (range: 5–21 years; S1) and after a mean of 18 years (range: 13–28 years; S2) (10). At baseline, the risk of testosterone deficiency was higher in the orchiectomy-alone group (*n* = 69; OR = 4.7) than for radiotherapy (*n* = 130; OR = 2.6) and chemotherapy (*n* = 108; OR = 1.9), when compared to controls. At S2, the risk of low testosterone levels was significantly higher in patients receiving chemotherapy (OR = 5.2) than in those treated with radiotherapy (OR = 3.3) or surgery alone (OR = 2). Similar results were found for the risk of high LH serum levels. Therefore, in contrast to surgery alone, both groups receiving radio- and chemotherapy (with a higher effect in the latter) had a lower Leydig cell function with time. In addition, the cumulative platinum dose was significantly associated with the risk of increasing LH levels for each cycle. These results suggest a functional reserve decrement in testosterone production of the remaining testis, which makes testicular tumor survivors vulnerable to the aging-related decline of Leydig cell function (late-onset hypogonadism). Furthermore, residual long-term serum platinum levels and the consequent chronic exposure of the testicular tissue may contribute to hypogonadism as well and may explain the reason why the group treated with chemotherapy has worse Leydig cell function (10).

In agreement with these findings, in a more than 5-year-long follow-up prospective study on 680 patients, low testosterone levels were found in 11% of the group of patients undergoing orchiectomy (*n* = 169), while a significantly higher portion of patients with low testosterone levels was found in patients receiving both radiotherapy and chemotherapy (37%, *n* = 81). Irradiated patients (*n* = 158) and those who received chemotherapy (*n* = 272) showed abnormally high LH levels in the 11% and in the 10% of cases, respectively. The results of this study confirmed that gonadal dysfunction is common in testicular tumor survivors even when managed with orchiectomy alone. Chemotherapy seems to result in an additional risk of testicular failure (24).

A meta-analysis of cohort studies definitively confirmed the occurrence of a higher risk for testosterone deficiency in TGCT patients treated with standard chemotherapy (≤4 platinum-based for chemotherapy cycle; OR 1.8), non-conventional chemotherapy (platinum-based combination chemotherapy with double dose of cisplatin, >4 cycles of platinum-based combination chemotherapy, or both chemotherapy and radiotherapy; OR 3.1), and radiotherapy (OR 1.6) when compared to patients with orchiectomy alone (22). The follow-up time of the studies included in this meta-analysis (22) ranged from only 2 months to 12 years, and some of them reported Leydig cell recovery in the years following the treatment. Accordingly, when patients are monitored for <5 years, the occurrence of hypogonadism is less frequently reported. In fact, a study carried out in 143 TGCT patients found a higher risk for hypogonadism in patients treated with radiotherapy or with three to four chemotherapy cycles when compared to adjuvant chemotherapy (≤2 cycles) at the 6 and 12th post-therapy month. Adjuvant chemotherapy consisted of no more than two cycles of combined therapy with bleomycin plus cisplatin plus etoposide or vinblastin, or carboplatin single administration, and it was offered to patients with a clinical stage I testicular tumor. High-dose chemotherapy consisted of three to four cycles, and it was administered to patients with more advanced disease. By contrast, no difference was found in further surveillance time points (24, 36, and 60 months). High doses of chemotherapy or radiotherapy seem to be, therefore, more harmful than the adjuvant chemotherapy on Leydig cell function, at least during the first-year post-treatment (14). This study also investigated whether any predictor of testosterone deficiency development in testicular tumor survivors does exist. Interestingly, while testicular volume, consistency, age, androgen receptor polymorphisms, and tumor stage have not been found to correlate with risk of hypogonadism, both mycrolithiasis in the remaining testis and the presence of low testosterone levels after orchiectomy but prior to any other treatment predicted the risk of developing hypogonadism (14). The reason why mycrolithiasis may somehow be associated with a higher risk of Leydig cell failure might be inherent to the possible existence of a tumor-dependent mechanism of Leydig cell damage. Testicular mycrolithiasis belongs to the TDS spectrum, the latter syndrome being considered to be involved in testicular tumor pathogenesis (26, 27).

**Figure 1:**
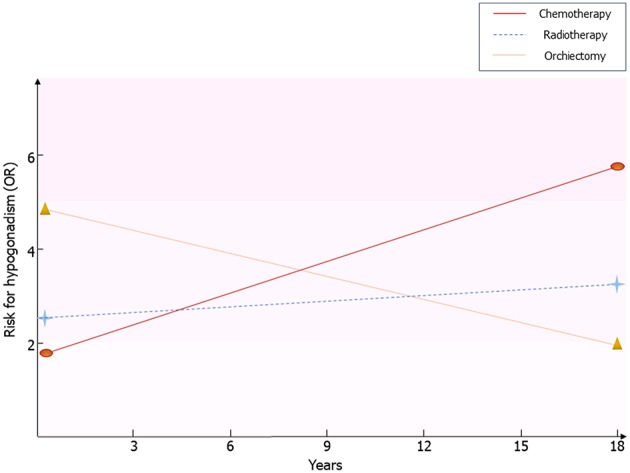
Risk for hypogonadism in testicular tumor survivors. Results coming from the available follow-up studies are resumed. Risk of hypogonadism has been calculated in comparison with healthy men. OR, odds ratio.

Framing together these results, Leydig cell vulnerability to chemotherapy and radiotherapy results in an impaired function in the first post-treatment year (14), with an apparent restoration of the Leydig cell function after at least 5 years from the treatment (14). The subsequent later decline of the function, due to subtler damage of Leydig cell function, seems to initially arise with a first phase of subclinical hypogonadism, consisting of increased LH and normal testosterone levels (13, 24), until the full onset of testosterone deficiency (10). This more likely happens in older patients, due to greater susceptibility of Leydig cells to the aging-induced damage in testicular tumor survivors, as previously suggested (10, 13) (Figure 1).

### Testicular Tumor Survivors: Long-Term Complications

#### Cardiovascular Diseases

The number of testicular tumor survivors has markedly increased through the decades. A multicenter study carried out on 1,214 testicular tumor patients treated with platinum-based chemotherapy has recently investigated the prevalence of adverse health outcomes (the so-called “Platinum Study”), in an attempt to assess long-term platinum-dependent toxicities. Mean age of patients was 37 years (range: 18–74 years), and the mean time from chemotherapy completion was 4.2 years (range: 1–30 years). Hypertension, peripheral artery disease, and a thromboembolic event were reported in the 9.4, 4.6, and 7.2% of cases, respectively. Coronary artery disease and cardiovascular events such as transient ischemic attack and stroke were negligible (1.6, 0.7, and 0.5%, respectively). Interestingly, the Reynaud phenomenon occurred in 33.4% of patients (25). In support of these findings, ongoing endothelial cell and vascular damage and hypertension could be related to the long-term serum platinum levels (28, 29).

The 10-year cardiovascular risk assessed by the Framingham Risk Score (3%) and the Systemic Coronary Risk Evaluation (1.7%) algorithms was comparable to controls and was independent of the treatment (30). By contrast, a greater risk of developing cardiovascular disease was found after 10.2 years of observation in 992 testicular tumor survivors (31). Other studies confirmed these findings (32). Similarly, a 20-year follow-up study carried out in 990 testicular tumor survivors and 990 age-matched controls more recently found a 5.7-fold higher risk for coronary artery disease in patients treated with chemotherapy (BEP) alone (*n* = 364) compared with surgery alone (*n* = 206) and a 3.1-fold higher risk for myocardial infarction in survivors treated with chemotherapy alone compared with controls. Both groups of patients receiving chemotherapy and radiotherapy (*n* = 386) showed an increased prevalence of administration with antihypertensive and antidiabetic drugs compared with controls. Atherosclerosis was observed only in 8% of patients, despite an increased risk for atherosclerotic disease observed in the chemotherapy and radiotherapy groups (both single and combined administration) compared with surgery alone. The risk was greater in the case of combined chemotherapy and radiotherapy (33). Summary of available data from studies on cardiovascular risk factors and diseases in testicular cancer survivors is described in Table 3.

**Table 3:** Summary of available data from studies on cardiovascular risk factors and cardiovascular diseases in testicular cancer survivors.

**References**	**Study design**	**Total sample**	**Time of enrollment/Follow-up**	**Results**
Meinardi et al. (32)	Cross-sectional	87 patients (long-term survivors of metastatic testicular cancer treated with cisplatin-based chemotherapy) and 40 controls (affected by stage I testicular tumor treated with orchiectomy alone)	>10 years post-therapy	• An increased observed-to-expected ratio for coronary artery disease was found in patients compared to general male Dutch population • The 33% of patients showed impaired diastolic left ventricular function • Patients has higher blood pressure, total cholesterol, and triglycerides and were more insulin resistant compared to controls
Haugnes et al. (34)	Prospective	1,135 patients (225 were treated with orchiectomy alone, 446 with radiotherapy, 376 with a cumulative cisplatin dose ≤850 mg, 88 with a cumulative cisplatin dose >850 mg)and 1,150 controls	9–12 years	• Increased odds for metabolic syndrome in patients treated with chemotherapy (cisplatin >850 mg) compared both to the surgery group (OR 2.8) and controls • The cisplatin ≤850 mg group had higher odds for metabolic syndrome compared only to the surgery group (OR 2.1) • Patients treated with radiotherapy did not show increased odds compared to the surgery group
Huddart et al. (31)	Prospective	992 patients	10.2 years	• Increased risk for cardiac events was registered after chemotherapy alone (RR 2.59), radiotherapy alone (RR 2.40), and chemotherapy plus radiotherapy (RR 2.78)
Haugnes et al. (33)	Prospective	990 patients (206 were treated with orchiectomy alone, 386 with radiotherapy alone, 364 with chemotherapy alone, 34 with combined radiotherapy, and chemotherapy) and 990 controls (healthy subjects from general population)	19 years	• Radiotherapy alone (OR 2.3) and radiotherapy plus chemotherapy (OR 3.9) groups showed and increased prevalence of diabetes mellitus compared to controls • Chemotherapy group has a 5.7-fold higher risk for coronary artery disease compared to surgery alone and a 3.1-fold higher risk for myocardial infarction compared to controls
Willemse et al. (30)	Cross-sectional	255 patients and 360 controls	7.8 years post-therapy	• Patients treated with combined chemotherapy had a higher risk for metabolic syndrome compared to controls
de Haas et al. (35)	Retrospective	370 patients treated with chemotherapy	≥3 years post-therapy	• Metabolic syndrome was detected in the 25% of patients
Kerns et al. (25)	Cross-sectional	1,214 patients treated with cisplatin-based chemotherapy	≥1 year post-therapy	• Obesity occurred in the 41.7% of patients • Patients had a high risk for hyperlipidemia, hypertension, and diabetes (OR 9.8)

*OR, odds ratio; RR, risk rate*.

In summary, these findings suggest the presence of a greater risk of developing cardiovascular diseases in testicular tumor survivors, especially after chemotherapy. Two hypotheses have been proposed to explain this association. The direct one suggests a chemotherapy-induced damage at the endothelial level. The indirect hypothesis ascribes the risk to the increased incidence of cardiovascular risk factors, such as hypertension, dyslipidemia, metabolic syndrome, and diabetes, which, in turn, raise the susceptibility to cardiovascular diseases (28).

#### Metabolic Diseases

According to the findings of the “Platinum Study,” which investigated 1,214 testicular cancer survivors, the most frequent adverse outcome observed 4.2 years after chemotherapy completion was obesity, with a prevalence of 71.5%. Diabetes and hypertriglyceridemia rarely occurred (3 and 0.5%, respectively), and hypercholesterolemia was reported in 8% of cases (25).

A follow-up study (1998–2002) on 1,135 testicular tumor survivors younger than 60 years assessed the association between metabolic syndrome (the modified National Cholesterol Education Program definition was used) and type of testicular tumor treatment. The sample studied included patients treated with surgery alone (*n* = 225), radiotherapy (*n* = 446), and cumulative cisplatinum dose ≤850 mg (*n* = 376) and >850 mg (*n* = 88). A greater risk for metabolic syndrome was found in both groups of patients receiving chemotherapy compared with those who underwent to surgery alone. The group treated with the higher cisplatinum cumulative dose showed a greater risk compared to controls (*n* = 1150), even after adjusting for testosterone levels, thus suggesting that this risk is not dependent on hypogonadism but is due to cisplatinum-induced damage (34). However, other studies have shown that serum testosterone levels <15 nmol/L are associated with a greater risk for developing metabolic syndrome in testicular tumor survivors (35). Indeed, after a median follow-up of 5 years, testicular tumor survivors treated with chemotherapy showed a 2.2-fold higher risk of developing metabolic syndrome compared with controls, whereas the risk increased up to 4.1-fold in survivors whose testosterone levels were <15 nmol/L. Furthermore, among the entire cohort of patients, overweight, and hypercholesterolemia were both found in 24% of cases (35). Similar findings were also reported in a study showing a higher risk of metabolic syndrome in a cohort of 255 testicular tumor survivors 7.8 years after chemotherapy (36). The risk was 2.5-fold higher in survivors with hypogonadism (30).

In conclusion, several reports have found the presence of different dysmetabolic diseases [obesity, metabolic syndrome, and diabetes mellitus (DM)], hypogonadism, and other cardiovascular risk factors. Their early diagnosis and proper treatment are of paramount relevance to lower the long-term cardiovascular risk in testicular tumor survivors.

#### Bone Density

The occurrence of a decreased bone mineral density (BMD) has been suggested in testicular tumor survivors. A prospective study on 63 germ-cell testicular tumor patients (mean age: 33 years; range: 16–70 years) showed a significant bone loss (lumbar spine BMD: −1.52%; total hip BMD: −2.05%) after 1 year from combination chemotherapy in patients with metastatic testicular tumor (*n* = 36), with no sign of recovery up to 5 years of follow-up. The decrease in BMD was not related with gonadal function, vitamin D levels, cisplatin cumulative dose, or corticosteroid administration. In contrast, stage I patients with no evidence of metastasis, treated with surgery alone or combined with a single dose of adjuvant chemotherapy, did not show any significant difference in BMD (37). In addition, lower BMD was observed in testicular germ-cell tumor patients treated with unilateral orchiectomy (*n* = 125) compared to age-matched controls (*n* = 41), despite the absence of hypogonadism (38). A cross-sectional study in 199 long-term testicular tumor survivors evaluated after a mean of 7.4 years from unilateral orchiectomy and in 45 newly diagnosed testicular tumor patients 3 months after orchiectomy showed an increased prevalence of mild and moderate vertebral fractures (40.2 and 31.1%, respectively) by the Genant's semi-quantitative method, independently of BMD, type of treatment, and gonadal function (39). Furthermore, osteopenia or osteoporosis was found in 43–51% of cases among a cohort of 1,249 long-term testicular tumor survivors. Hypogonadism more frequently occurred in patients with reduced BMD, but all survivors with osteopenia or osteoporosis showed lower testosterone levels. The patients treated with radiotherapy did not show a significantly worse BMD compared with those who received chemotherapy or surgery alone (23). Accordingly, the 9-year-long follow-up in 91 testicular tumor survivors (mean age: 31 years) revealed a significantly 6–8% lower hip BMD in both untreated and treated hypogonadal survivors compared to eugonodal ones and a significant 8% lower spinal BMD in untreated hypogonadal compared to eugonodal survivors (40), thus suggesting the increased risk of impaired bone health in hypogonadal testicular tumor survivors. By contrast, a single study on only 39 testicular tumor (TT) patients after a follow-up time ranging from 5 to 28 years did not find abnormal BMD in patients treated with surgery alone or with chemotherapy (41). Summary of available data from studies on bone mineralization in testicular cancer survivors is described in Table 4.

**Table 4:** Summary of available data from studies on bone mineralization in testicular cancer survivors.

**References**	**Study design**	**Total sample**	**Time of enrollment/Follow-up**	**Results**
Murugaesu et al. (41)	Cross-sectional	39 patients	5–28 years	• Orchiectomy alone or orchiectomy plus chemotherapy predisposed to osteoporosis
Willemse et al. (39)	Cross-sectional	199 patients treated with chemotherapy and 45 newly diagnosed patients within 3 months after orchiectomy	7.4 years post-treatment	• The 25.8% of patients had Z-score between −1 and −2 SD, the 12% of patients has Z-score below −2 SD • Moderate and severe vertebral fractures were observed in 13.6% of cured-long term survivors and in 15.6% of newly diagnosed patients
Foresta et al. (38)	Case–control	125 normotestosteronemic patients treated with orchiectomy and 41 controls	NR	• Vitamin D serum levels was lower in patients than in controls • The 23.8% of patients had Z-score below −2 SD
Willemse et al. (37)	Prospective	63 patients (27 were treated with orchiectomy, 36 received chemotherapy)	5 years post-treatment	• Normal values of bone mineral density were detected in patients treated with orchiectomy only • Significant bone loss was observed in patients receiving chemotherapy
Isaksson et al. (40)	Case–control	91 patients and 91 controls	9.3 years	• Compared to eugonodal patients, patients with hypogonadism receiving or not testosterone replacement therapy had 6–8% lower hip bone mineral density
Ondrusova et al. (23)	Cross-sectional	1,249 patients (313 treated with orchiectomy, 665 with chemotherapy, 271 with radiotherapy)	35 years post-treatment	• Osteopenia or osteoporosis occurred in 136 patients treated with orchiectomy, 298 patients treated chemotherapy, and 139 patients treated with radiotherapy

*NR, not reported; SD, standard deviation*.

In conclusion, vertebral fractures and impaired BMD occur in testicular tumor survivors, but it is still unclear whether it is related to hypogonadism or to cancer therapy-induced bone damage. Osteological examination should be considered in the follow-up of these patients.

## Sexual Function

Sexual dysfunction is often experienced by testicular tumor survivors. The available evidence on this topic is summarized in Table 5.

**Table 5:** Summary of available data from studies on sexual dysfunction in testicular tumor survivors.

**References**	**Study design**	**Total sample**	**Time of enrollment/Follow-up**	**Methods**	**Results**
Nazareth et al. (42)	Meta-analysis	709 patients from six controlled and 337 patients from seven uncontrolled studies	Up to 2 years post-treatment	Self-reported or structured questionnaire	• Significantly reduced or absent orgasm (OR 4.6) in patients vs. controls • ED (OR 2.5) in patients vs. controls • Ejaculatory dysfunction (OR 28.6) in patients vs. controls
Eberhard et al. (43)	Case–control	129 patients and 916 age-matched controls	3–5 post-treatment	NR	• Patients have a higher risk for ED (OR 3.3) and low sexual desire (OR 6.7) 3 to 5 years after TT treatment
Tuinman et al. (44)	Prospective	93 patients	1, 3, and 12 months after orchiectomy	IIEF	• Orgasm and EF decreased 3 months after orchiectomy and restored 1 year later • Singles reported more sexual problems compared to partnered patients and to controls
Tasdemir et al. (45)	Case–control	27 patients treated with chemotherapy and controls	>3 years post-treatment	IIEF-15	• The IIEF-15 score was significantly lower in patients vs. controls
Kim et al. (46)	Case–control	246 patients vs. 236 age-matched controls	>5 years post-treatment	BSFI	• Patients scored lower on sex drive, erection, ejaculation, and problem assessment vs. controls • Chemotherapy or radiotherapy increased the risk for sexual dysfunction vs. controls • Surgery-only treatment did not increase the risk for sexual dysfunction vs. controls
Pühse et al. (47)	Cross-sectional	539 patients	After completion of oncologic therapy	IIEF-15BSFI	• ED occurred in the 31.5% of patients (due to inability to maintain erection on the 24.4% of cases) • Ejaculatory disorders (premature, delayed, retrograde, anejaculation) were reported in the 84.9% of cases • The 32.4% of cases experienced reduced intensity of orgasm • The 95.4% of cases referred reduced overall sexual satisfaction
Bumbasirevic et al. (48)	Cross-sectional	202 patients	47.3 ± 26.8 months	SF 36	• ED was reported by the 20.8% of patients • Loss of desire was reported by the 17.3% of patients • Ejaculatory dysfunction was reported by the 25.7% of patients
Alacacioglu et al. (49)	Case–control	41 patients vs. 38 controls	NR	GRISS	• Patients scored lower on satisfaction, erection, avoidance, and touch vs. controls
Wortel et al. (50)	Prospective	161 patients	Prior to radiotherapy and after 3 and 6 months	Dutch questionnaire	• ED was found in the 23% of patients
Capogrosso et al. (51)	Prospective cross-sectional	143 patients	86 months	IIEF-15	• ED occurs in the 25.5% of patients, being severe in the 11.2% of cases • Mean time of EF recovery is 60, 60, and 70 months after chemotherapy, radiotherapy, and RPLND, respectively • Adjuvant RT is an independent predictor of no recovery of normal EF
Catanzariti et al. (52)	Prospective	67 patients with prosthesis implantation	Before and 6 months after orchiectomy	IIEF-15PEDT	• No change in questionnaire scores • The 22.4% of patients were dissatisfied about the prosthesis
Dimitropoulos et al. (53)	Prospective	53 patients treated with post-chemotherapy full bilateral non-nerve sparing RPLND	Before and 3 months after operation	IIEF-15	• No change in questionnaire scores • Orgasmic function and intercourse and overall sexual satisfaction were significantly impaired post-operatively
Bandak et al. (54)	Cross-sectional	2,260 patients (1,098) treated by orchiectomy alone, 788 with chemotherapy alone or post-chemotherapy RPLND, 300 with radiotherapy, 74 receiving more than one line of treatment	17 years	IIEF-15	• The risk for ED was higher in chemotherapy (OR 1.5), post-chemotherapy RPLND (OR 2.1), radiotherapy (1.7), and more than one line of treatment (OR 3.2) groups vs. orchiectomy alone group • Orgasmic dysfunction was associated with radiotherapy, post-chemotherapy RPLND, and more than one line of treatment
Kerns et al. (25)	Cross-sectional	1,214 patients treated with chemotherapy or post-chemotherapy RPLND	4.2 years post-treatment (range: 1–30 years)	Self-reported	• ED occurs in 28.4% of patients

*BSFI, Brief Sexual Function Inventory; BSF, Brief Sexual Function Inventory; ED, erectile dysfunction; EF, erectile function; GRISS, Golombok–Rust Inventory of Sexual Satisfaction; NR, not reported; RPLND, retroperitoneal lymph node dissection; SF, short form; TT, testicular tumor*.

### Erection

A number of reports have evaluated the erectile function among testicular tumor survivors (36, 46). A multicenter study encompassing more than 1,200 survivors reported a 4.2-fold higher risk of erectile dysfunction (ED) in testicular tumor survivors compared with controls (25). The prevalence of ED has been esteemed to range from 30 to 40% (45–47, 55) in testicular tumor survivors, mainly assessed by the International Index of Erectile Function (IIEF) questionnaire and largely due to the incapacity to maintain the erection (47).

A meta-analysis of controlled studies found a ~2.5-fold greater risk of ED up to 2 years after treatment (42). Data from a longitudinal study showed a median time of erectile function recovery of 60, 60, and 70 months in patients receiving radiotherapy, chemotherapy, and RPLND, respectively, after a ~7.5-year-long follow-up in 143 Caucasian-European testicular tumor survivors. Only adjuvant radiotherapy emerged as an independent predictor of non-recovery (51). Accordingly, the Childhood Cancer Survivor Study indicated a negative impact of radiotherapy on erectile function, since a ≥10-Gr testicular irradiation dose was associated with a greater risk of ED (RR 3.55) among a cohort of 1,622 male cancer survivors (mean age: 37.2 years) (56). However, chemotherapy is also capable of negatively influencing sexual function. Data from a controlled study reported worse scores at the IIEF-15 and the Beck Anxiety questionnaire in patients receiving chemotherapy more than 3 years before evaluation compared to the age-matched controls who did not undergo to chemotherapy. The absence of any significant difference in serum gonadotropin and testosterone levels between the two groups suggests that the greater risk of ED is independent from hypogonadism. However, the small sample size (*n* = 27) limits the reliability of the study results (45). In addition, a longitudinal, cross-sectional study from 202 Serbian testicular tumor survivors followed-up for at least 1 year after platinum-based chemotherapy reported ED in 20.8% of cases (using the SF questionnaire). No patient of this cohort underwent testicular prosthesis implantation due to their socioeconomic background (48). Testicular prosthesis does not seem to affect sexual function *per se*; a part patients complain about is its consistence (52).

These results suggest that the type of testicular tumor has clear implications in the erectile function. Orchiectomy alone may be preferred to other treatment strategies, when possible. Moreover, following an initial post-therapy damage, the erectile function seems to re-establish itself 6 years after the treatment (51). However, a longer time of observation suggests different conclusions. Very recently, a comprehensive prospective study carried out in a cohort of 2,260 testicular tumor survivors reported an increased risk of ED after a 17-year-long follow-up. In greater detail, the study population included 1,098 patients who underwent orchiectomy alone, 788 treated with chemotherapy (BEP) alone or post-retroperitoneal surgery, 300 patients treated with radiotherapy, and 74 receiving more than one treatment. ED was assessed by the IIEF-15 questionnaire. Compared to orchiectomy alone, the survey showed an increased risk of ED in patients who received chemotherapy (OR 1.5), chemotherapy plus post chemotherapy testicular surgery (OR 2.1), RT (OR 1.7), or more than one type of treatment (OR 3.2) (54), thus showing that additional treatments negatively impact the erectile function. Accordingly, data from other reports agree with the worse impact of RPLND following chemotherapy on erectile function (57, 58).

### Orgasm and Ejaculation

About one third of testicular tumor survivors experience ejaculation dysfunction (45). In addition, a ~2.3 higher risk of impaired ejaculation has been reported in these patients compared with controls, being even higher (OR 3.06) in non-seminoma patients (47). A meta-analysis of controlled studies found a decreased or absent orgasmic sensation associated with ejaculatory dysfunction in testicular tumor survivors up to 2 years after the treatment (59). After 17 years of follow-up, orgasmic dysfunction seems to persist and to associate with radiotherapy, chemotherapy plus post-chemotherapy RLND, and more than one line of treatment in 2,260 testicular tumor survivors (52).

Treatment options may also influence the ejaculatory function in testicular tumor survivors. Chemotherapy showed a greater risk of delayed ejaculation compared to radiotherapy and surgery (46). Full bilateral, non-nerve-sparing RLND may associate with ejaculatory disorders compared to other treatments, probably due to a damage on the sympathetic nerve fibers that control ante-grade ejaculation (53). Accordingly, despite no difference in erectile function following post-chemotherapy RLND observed, orgasmic function and satisfaction were significantly impaired post-operatively, compared to pre-operative function in a cohort of 53 patients (53).

## Conclusion

**Figure 2:**
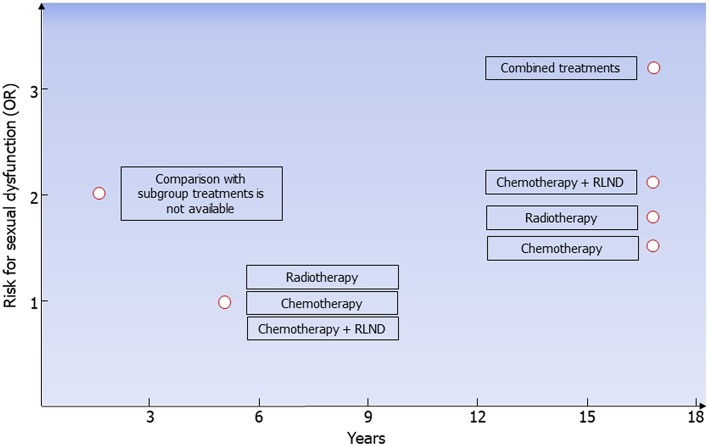
Risk for erectile dysfunction in testicular tumor survivors. According to data coming from all the available follow-up studies, risk for erectile dysfunction (ED) is higher 2 years after treatment in testicular tumor survivors. At the fifth year following radiotherapy, chemotherapy, or chemotherapy plus retroperitoneal lymph node dissection (RLND), the erectile function is apparently restored. The risk for ED is higher in patients treated with chemotherapy (OR 1.5), radiotherapy (OR 1.7), chemotherapy plus RLND (OR 2.1), and combined treatments (OR 3.2) compared to those treated with surgery only.

Since the introduction of platinum-based chemotherapy and radiotherapy, the 10-year survival rate of patients with testicular tumor has exceeded 97%. The choice of treatment, especially in stage I, where treatment options include surveillance, adjuvant chemotherapy, or adjuvant radiotherapy (6), should take into consideration the risk for long-term complications. Longitudinal studies have revealed a higher negative impact of chemotherapy on Leydig cell function than radiotherapy or orchiectomy alone, leading to a higher risk for hypogonadism. Compared to orchiectomy alone, combined or high-dose chemotherapy and radiotherapy increase the risk for metabolic syndrome, DM, and cardiovascular events (Table 6). Furthermore, the long-term risk for ED is higher in patients treated with combined treatments, chemotherapy plus RPLND, radiotherapy, and chemotherapy compared to orchiectomy alone (Figure 2). On this account, orchiectomy and clinical surveillance should be preferred. Finally, management of testicular tumor survivors should include the evaluation of gonadal function, cardiovascular and metabolic profiles, BMD, and sexual function to timely detect any possible impairment.

**Table 6:** Risk for cardiovascular and metabolic complications in testicular tumor survivors.

	**Chemotherapy**	**Radiotherapy**
Metabolic syndrome	• Increased for combined therapy or high-dose cisplatin-based chemotherapy • Non-increased for low dose cisplatin-based chemotherapy	Non-increased
Diabetes mellitus	• Increased for combined therapy	Increased
Cardiovascular events	• Increased for combined therapy	Increased

*Combined therapy included PVB (cisplatin, vinblastine, and bleomycin) and BEP (bleomycin, etoposide, and cisplatin) schemes; low-dose cisplatin-based chemotherapy: ≤ 850 mg cumulative dosage; high-dose cisplatin-based chemotherapy: >850 mg cumulative dosage*.

## Author Contributions

RCa and RCo conceived the work and wrote the paper. RCa, FB, YD, GB, and MC identified the articles. AD and SL revised the paper critically and gave final approval. All authors read and approved the final manuscript.

### Conflict of Interest Statement

The authors declare that the research was conducted in the absence of any commercial or financial relationships that could be construed as a potential conflict of interest.
