# Corrigendum: Hypogonadism and Sexual Dysfunction in Testicular Tumor Survivors: A Systematic Review

**DOI:** 10.3389/fendo.2019.00393

**Published:** 2019-06-19

**Authors:** Sandro La Vignera, Rossella Cannarella, Ylenia Duca, Federica Barbagallo, Giovanni Burgio, Michele Compagnone, Andrea Di Cataldo, Aldo E. Calogero, Rosita A. Condorelli

**Affiliations:** ^1^Section of Endocrinology, Department of Clinical and Experimental Medicine, University of Catania, Catania, Italy; ^2^Unit of Pediatric Hematology and Oncology, Department of Clinical and Experimental Medicine, University of Catania, Catania, Italy

**Keywords:** hypogonadism, testicular tumor, testosterone, sexual dysfunction, cardiovascular risk

An author name was incorrectly spelled as Andrea Di (first name) Cataldo (last name). The correct spelling is Andrea (first name) Di Cataldo (last name). The authors apologize for this error and state that this does not change the scientific conclusions of the article in any way. The original article has been updated.

